# Supplementary material for: The Evolution and Characterization of the RNA Interference Pathways in Lophotrochozoa
Source: Genome Biol Evol. 2024 May 7;16(5):evae098. doi: 10.1093/gbe/evae098 (PMC11114477; doi:10.1093/gbe/evae098)
Supplement: evae098_Supplementary_Data [file evae098_supplementary_data.zip › Supplementary_materials.zip!/selected_miRNAs/NoGe_selected_miRNAs/mirdeep_pdf_files/NMRB01000231.1_Notospermus_geniculatus_strain_Ushimado_scaffold231,_whole_genome_shotgun_sequence_7809.pdf]

Provisional ID	:	NMRB01000231.1_Notospermus_geniculatus_strain_Ushimado_scaffold231,_whole_genome_shotgun_sequence_7809
Score total	:	55280.5
Score for star read(s)	:	3.9
Score for read counts	:	55271.8
Score for mfe	:	1.8
Score for randfold	:	0
Score for cons. seed	:	3
Total read count	:	108425
Mature read count	:	108131
Loop read count	:	0
Star read count	:	294

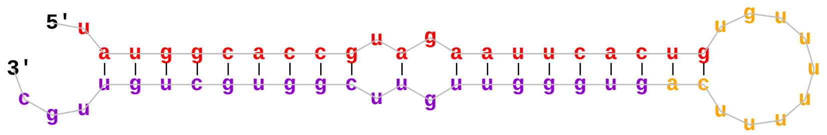

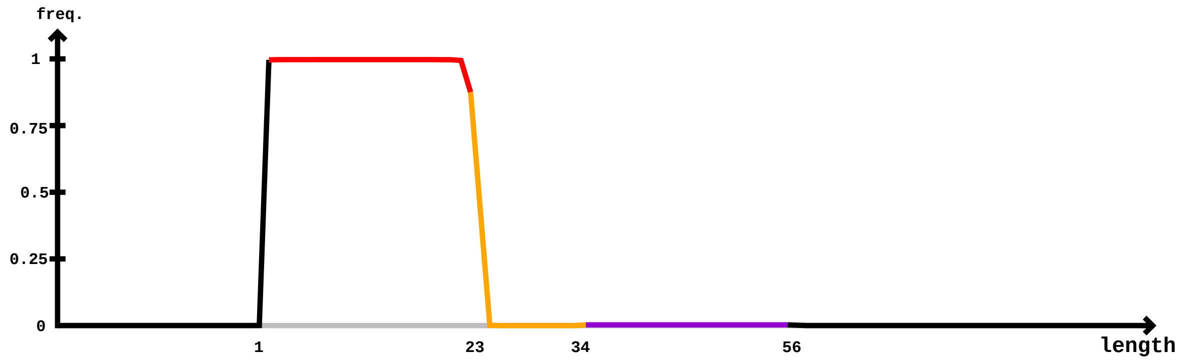

Mature Star

```
5'- ucaucucugcuuacaagagcuauggcaccguagaauucacuguguuuuuuucagugggguuguucggugcuguugcuuuugauguagauagcugcuguugucacuugugauuc  -3'   obs
    ucaucucugcuuacaagagcuauggcaccguagaauucacuguguuuuuuucagugggguuguucggugcuguugcuuuugauguagauagcugcuguugucacuugugauuc         exp
    .......(((.(.(((((((.((((((((.(.((((((((.........)))))))).).))))))))).))))))))).)))((((((....)))))).........  reads   mm   sample
    ..................cuauggcaccguagaauucacu.........................................................................  1       0    Fm3
    ..................cuauggcaccguagaauucacug........................................................................  1       0    Fm3
    ...................uauggcaccguagaauuc............................................................................  4       0    Fm3
    ...................uauggcaccguagaauuA............................................................................  4       1    Fm3
    ...................uauggcaccguagaauuca...........................................................................  4       0    Fm3
    ...................uauggcaccguagaauucaA..........................................................................  1       1    Fm3
    ...................uauggcaccguagaauucac..........................................................................  15      0    Fm3
    ...................uauggcaccguagaauuUac..........................................................................  1       1    Fm3
    ...................uaugAcaccguagaauucacu.........................................................................  1       1    Fm3
    ...................uauggcaccgCagaauucacu.........................................................................  1       1    Fm3
    ...................uauggcaccguagaauuUacu.........................................................................  4       1    Fm3
    ...................uauggcacUguagaauucacu.........................................................................  6       1    Fm3
    ...................uauggcaccguagaauGcacu.........................................................................  1       1    Fm3
    ...................uauggcUccguagaauucacu.........................................................................  2       1    Fm3
    ...................uauggcaccguagaauAcacu.........................................................................  4       1    Fm3
    ...................uauggcaccguagaauucacu.........................................................................  547     0    Fm3
    ...................uauggcaccguagaauuAacu.........................................................................  6       1    Fm3
    ...................uauggcaccguagaauuGacu.........................................................................  4       1    Fm3
    ...................uauggcGccguagaauucacu.........................................................................  2       1    Fm3
    ...................uauggcaccguagaauucacug........................................................................  1960    0    Fm3
    ...................uauggcaccguagaauuUacug........................................................................  11      1    Fm3
    ...................uaGggcaccguagaauucacug........................................................................  1       1    Fm3
    ...................uauggcaccguagaauuAacug........................................................................  10      1    Fm3
    ...................uauggcaccguagCauucacug........................................................................  1       1    Fm3
    ...................uauggcaccguagaCuucacug........................................................................  1       1    Fm3
    ...................uauggcGccguagaauucacug........................................................................  3       1    Fm3
    ...................uauggcaccguagaauucacAg........................................................................  1       1    Fm3
    ...................uaugUcaccguagaauucacug........................................................................  1       1    Fm3
    ...................uauggcaccguagaauCcacug........................................................................  6       1    Fm3
    ...................uauggcaccguagaauGcacug........................................................................  5       1    Fm3
    ...................uauggcaccguagUauucacug........................................................................  1       1    Fm3
    ...................uauggcacUguagaauucacug........................................................................  3       1    Fm3
    ...................uauggcaccguaUaauucacug........................................................................  1       1    Fm3
```

Mature **Star**

```
ucaucucugcuuacaagagcuauggcaccguagaauucacuguguuuuuuucaguggguuguucggugcuguugcuuuugauguagauagcugcuguugucacuugugauuc
....................uauggcaccguagaauucGcug...................................................................... 1 1 Fm3
....................uauggcaAcguagaauucacug...................................................................... 2 1 Fm3
....................uauggcaccguagaauuGacug...................................................................... 5 1 Fm3
....................uauggcaccgGagaauucacug...................................................................... 1 1 Fm3
....................uauggcaccguagaauucacuU...................................................................... 1 1 Fm3
....................uauggcaccguagaaCucacug...................................................................... 1 1 Fm3
....................uauggcaccguagaauAcacug...................................................................... 5 1 Fm3
....................uaAggcaccguagaauucacug...................................................................... 2 1 Fm3
....................Cauggcaccguagaauucacug...................................................................... 1 1 Fm3
....................uauggUaccguagaauucacug...................................................................... 4 1 Fm3
....................Nauggcaccguagaauucacug...................................................................... 1 1 Fm3
....................uauggcaccAuagaauucacug...................................................................... 1 1 Fm3
....................uauggcaccguCgaauucacug...................................................................... 2 1 Fm3
....................uauggcaccguagaauucacuA...................................................................... 6 1 Fm3
....................uauggcaccgAagaauucacug...................................................................... 7 1 Fm3
....................uauggcaccguagGauucacug...................................................................... 1 1 Fm3
....................Nauggcaccguagaauucacugu..................................................................... 1 1 Fm3
....................uauggcaccguagaauucacugu..................................................................... 168 0 Fm3
....................uauggcaccguagaauucacugA..................................................................... 26 1 Fm3
....................uauggcacUguagaauucacugu..................................................................... 2 1 Fm3
....................uauggcaccguagaauucacuguU.................................................................... 8 1 Fm3
....................uauggcaccguagaauucacuguA.................................................................... 1 1 Fm3
.....................auggcaccguagaauucacu....................................................................... 1 0 Fm3
.....................auggcaccguagaauucacuU...................................................................... 1 1 Fm3
.....................auggcaccguagaauucacug...................................................................... 4 0 Fm3
.....................auggcaccguagaauucacugA..................................................................... 1 1 Fm3
.....................auggcaccguagaauucacuguA.................................................................... 1 1 Fm3
.....................................................guggguuguucggugcuguug...................................... 3 0 Fm3
.....................................................guggguuguucggugcuguugc..................................... 3 0 Fm3

.caucucugcuuacaagagc............................................................................................ 1 0 Fm1
...................cuauggcaccguagaauucacu....................................................................... 1 0 Fm1
....................uauggcaccguagaauuc.......................................................................... 3 0 Fm1
....................uauggcaccguagaauucac........................................................................ 8 0 Fm1
....................uauggcaccguagaauucacu....................................................................... 1315 0 Fm1
....................uauggcaccUuagaauucacu....................................................................... 1 1 Fm1
....................uauggcaUcguagaauucacu....................................................................... 1 1 Fm1
....................uauggcaccguagaauGcacu....................................................................... 3 1 Fm1
....................uauggcaccguaCaauucacu....................................................................... 1 1 Fm1
....................uauggcaccguagaauAcacu....................................................................... 2 1 Fm1
....................uauggcaccgCagaauucacu....................................................................... 2 1 Fm1
....................uUuggcaccguagaauucacu....................................................................... 2 1 Fm1
....................uaugUcaccguagaauucacu....................................................................... 2 1 Fm1
....................uauggcacUguagaauucacu....................................................................... 1 1 Fm1
....................uauggcaccgGagaauucacu....................................................................... 4 1 Fm1
....................uauggcaccguagaauuUacu....................................................................... 5 1 Fm1
....................uauggcaccgAagaauucacu....................................................................... 3 1 Fm1
....................uauggcaccguagaauucacG....................................................................... 2 1 Fm1
....................uauggcGccguagaauucacu....................................................................... 4 1 Fm1
....................uauggcaccguagaauCcacu....................................................................... 1 1 Fm1
....................uauggcaccguagaauuGacu....................................................................... 3 1 Fm1
....................uauggcaccguagaaCucacu....................................................................... 2 1 Fm1
....................uauggcaccguagaauuAacu....................................................................... 10 1 Fm1
....................uauggcaccguagaauucacug...................................................................... 2733 0 Fm1
....................uauUgcaccguagaauucacug...................................................................... 2 1 Fm1
....................uauggcaccguagaauucacuA...................................................................... 9 1 Fm1
....................uauggcaccguagaauCcacug...................................................................... 7 1 Fm1
....................uauggcacUguagaauucacug...................................................................... 1 1 Fm1
....................uUuggcaccguagaauucacug...................................................................... 1 1 Fm1
....................uaCggcaccguagaauucacug...................................................................... 2 1 Fm1
....................uauggcaccguagaGuucacug...................................................................... 1 1 Fm1
....................uauggcaccguagaauGcacug...................................................................... 8 1 Fm1
....................uauggcaccguagaauuAacug...................................................................... 22 1 Fm1
....................uauggcaccguCgaauucacug...................................................................... 3 1 Fm1
....................uauggUaccguagaauucacug...................................................................... 1 1 Fm1
....................uauggcCccguagaauucacug...................................................................... 1 1 Fm1
....................uauggcaccguagUauucacug...................................................................... 1 1 Fm1
....................uauggcaccguagaauucacGg...................................................................... 12 1 Fm1
....................uauggcaccgCagaauucacug...................................................................... 2 1 Fm1
....................uaugUcaccguagaauucacug...................................................................... 1 1 Fm1
```

```
                   Mature                Star
ucaucucugcuuacaagagcuauggcaccguagaauucacuguguuuuuuucaguggguuguucggugcuguugcuuuugauguagauagcugcuguugucacuugugauuc
....................uauggcaccguagaauucUcug......................................................................  1     1  Fm1
....................uauggcaccguagaauucacuU......................................................................  4     1  Fm1
....................uGuggcaccguagaauucacug......................................................................  1     1  Fm1
....................uauggcaccguagaauuUacug......................................................................  13    1  Fm1
....................uauggcaccgGagaauucacug......................................................................  4     1  Fm1
....................uauggcaccguagaauucacuC......................................................................  8     1  Fm1
....................uauggcaccAuagaauucacug......................................................................  1     1  Fm1
....................uauggcaccguGgaauucacug......................................................................  2     1  Fm1
....................uauggcaccguagaauuGacug......................................................................  5     1  Fm1
....................uauggcaccguagaauucaGug......................................................................  1     1  Fm1
....................uauggcaccgAagaauucacug......................................................................  3     1  Fm1
....................uauggGaccguagaauucacug......................................................................  1     1  Fm1
....................uauggcaccguagaauucGcug......................................................................  1     1  Fm1
....................uauggcaccguagaauAcacug......................................................................  8     1  Fm1
....................uauggcaccguagCauucacug......................................................................  1     1  Fm1
....................uauggcaccguagaauuAacugu.....................................................................  1     1  Fm1
....................uauggcaccguagaauucacugu.....................................................................  173   0  Fm1
....................uauggcaccUuagaauucacugu.....................................................................  1     1  Fm1
....................uauggcaccguagaauucacugG.....................................................................  3     1  Fm1
....................uauggcaccguagaauucacugA.....................................................................  56    1  Fm1
....................uauggcaccguagaauuGacugu.....................................................................  2     1  Fm1
....................uauggcaccguagaauucacugC.....................................................................  3     1  Fm1
....................uauggUaccguagaauucacugu.....................................................................  1     1  Fm1
....................uauggcaccguGgaauucacugu.....................................................................  1     1  Fm1
....................uauggcaccguagaauuUacugu.....................................................................  1     1  Fm1
....................uauggcaccguagaauucUcugu.....................................................................  1     1  Fm1
....................uaCggcaccguagaauucacugu.....................................................................  1     1  Fm1
....................uauggcaccguagaauucacuguA....................................................................  1     1  Fm1
....................uauggcaccguagaauucacuguU....................................................................  3     1  Fm1
.....................auggcaccguagaauucacug......................................................................  1     0  Fm1
......................uggcaccguagaauucacu.......................................................................  1     0  Fm1
.....................................................guggguuguucggugcCguugc.....................................  1     1  Fm1
.....................................................guggguuguucggugcuguugc.....................................  10    0  Fm1

...................cuauggcaccguagaauucacu.......................................................................  6     0  Fm2
....................uauggcaccguagaauuA..........................................................................  9     1  Fm2
....................uaCggcaccguagaauucacu.......................................................................  1     1  Fm2
....................uauggcaccguagGauucacu.......................................................................  1     1  Fm2
....................uauggcaccguagaauucacG.......................................................................  9     1  Fm2
....................uCuggcaccguagaauucacu.......................................................................  2     1  Fm2
....................uauggcaccguagaauuGacu.......................................................................  11    1  Fm2
....................uGuggcaccguagaauucacu.......................................................................  2     1  Fm2
....................uauggcaccguGgaauucacu.......................................................................  2     1  Fm2
....................uauggcaccgGagaauucacu.......................................................................  1     1  Fm2
....................Nauggcaccguagaauucacu.......................................................................  1     1  Fm2
....................uauAgcaccguagaauucacu.......................................................................  2     1  Fm2
....................Aauggcaccguagaauucacu.......................................................................  1     1  Fm2
....................uauggcaccguagaauucacu.......................................................................  2311  0  Fm2
....................uauggcaccgCagaauucacu.......................................................................  4     1  Fm2
....................uauggcaccguagaaCucacu.......................................................................  4     1  Fm2
....................uauggcaccguagUauucacu.......................................................................  1     1  Fm2
....................uauggcaccguagaauuAacu.......................................................................  23    1  Fm2
....................uauggUaccguagaauucacu.......................................................................  1     1  Fm2
....................uauggcaccguagaauucacA.......................................................................  1     1  Fm2
....................uaGggcaccguagaauucacu.......................................................................  1     1  Fm2
....................uauggcaccAuagaauucacu.......................................................................  1     1  Fm2
....................uauggcaccguagaauAcacu.......................................................................  5     1  Fm2
....................uauggcaccguagaauuUacu.......................................................................  9     1  Fm2
....................uauggcaccgAagaauucacu.......................................................................  4     1  Fm2
....................uauggcacUguagaauucacu.......................................................................  36    1  Fm2
....................uauggcaccguagaauCcacu.......................................................................  5     1  Fm2
....................uauggcaccguagaauGcacu.......................................................................  2     1  Fm2
....................uauggNaccguagaauucacu.......................................................................  1     1  Fm2
....................Cauggcaccguagaauucacu.......................................................................  4     1  Fm2
....................uauggcaccgAagaauucacug......................................................................  7     1  Fm2
....................uCuggcaccguagaauucacug......................................................................  2     1  Fm2
....................uauggcaccguagaauucacuA......................................................................  28    1  Fm2
....................uauggcaccguagaauucacCg......................................................................  1     1  Fm2
....................uauggcacAguagaauucacug......................................................................  1     1  Fm2
....................Nauggcaccguagaauucacug......................................................................  3     1  Fm2
```

Mature **Star**

```
ucaucucugcuuacaagagcuauggcaccguagaauucacuguguuuuuuucagugguuguucggugcuguugcuuuugauguagauagcugcuguugucacuugugauuc
....................uauggcNccguagaauucacug.....................................................................  1     1   Fm2
....................uauggcaccgCagaauucacug.....................................................................  5     1   Fm2
....................uauggcaccgGagaauucacug.....................................................................  2     1   Fm2
....................uauggcaccguagaauAcacug.....................................................................  5     1   Fm2
....................uauggcaccguagaauucUcug.....................................................................  1     1   Fm2
....................uauggcaccguagaGuucacug.....................................................................  1     1   Fm2
....................uauggcaccguagaauucacGg.....................................................................  4     1   Fm2
....................uauggcaccguCgaauucacug.....................................................................  3     1   Fm2
....................uauggcaccguagaauucacug.....................................................................  2910  0   Fm2
....................uGuggcaccguagaauucacug.....................................................................  2     1   Fm2
....................uauggcaccguagaauuAacug.....................................................................  19    1   Fm2
....................uauggcaccguagaauGcacug.....................................................................  8     1   Fm2
....................uauggcaccguagaauuGacug.....................................................................  12    1   Fm2
....................uauggcaccguagaauCcacug.....................................................................  15    1   Fm2
....................uauggcaccguagaauucacuU.....................................................................  1     1   Fm2
....................uauggcaccguaUaauucacug.....................................................................  1     1   Fm2
....................uauggcacUguagaauucacug.....................................................................  4     1   Fm2
....................uauUgcaccguagaauucacug.....................................................................  1     1   Fm2
....................uauggcaccguUgaauucacug.....................................................................  1     1   Fm2
....................uUuggcaccguagaauucacug.....................................................................  1     1   Fm2
....................uauggcGccguagaauucacug.....................................................................  1     1   Fm2
....................uauggcaUcguagaauucacug.....................................................................  1     1   Fm2
....................uaugAcaccguagaauucacug.....................................................................  1     1   Fm2
....................uauggcaccguagaauucCcug.....................................................................  1     1   Fm2
....................uauggcUccguagaauucacug.....................................................................  1     1   Fm2
....................uauggcaccguagaauuUacug.....................................................................  23    1   Fm2
....................uauggcaccAuagaauucacugu....................................................................  1     1   Fm2
....................uauggcaccguagaauucacugu....................................................................  293   0   Fm2
....................uauggcaccguagaauucacugC....................................................................  14    1   Fm2
....................uauggcaccguagaauGcacugu....................................................................  1     1   Fm2
....................uauggcaccguagaauuGacugu....................................................................  1     1   Fm2
....................uauggcaccguagaauucacugG....................................................................  41    1   Fm2
....................uauggcaccguagaauuUacugu....................................................................  1     1   Fm2
....................uauggcaccguagaauucacugA....................................................................  171   1   Fm2
....................uauggcaccguagGauucacugu....................................................................  1     1   Fm2
....................uauggcaccgCagaauucacugu....................................................................  1     1   Fm2
....................uauggcaccguagaauucacugug...................................................................  1     0   Fm2
....................uauggcaccguagaauucacuguU...................................................................  12    1   Fm2
....................uauggcaccguagaauucacuguA...................................................................  8     1   Fm2
.....................auggcaccguagaauucacu......................................................................  2     0   Fm2

....................uauggcaccguagaauuc.........................................................................  3     0   Mm3
....................uauggcaccguagaauuca........................................................................  3     0   Mm3
....................uauggcaccguagaauucac.......................................................................  36    0   Mm3
....................uauggcaccguagaauucacu......................................................................  783   0   Mm3
....................uauggcaccguagaauucacA......................................................................  2     1   Mm3
....................uauggcaccguagaauCcacu......................................................................  1     1   Mm3
....................uauggcGccguagaauucacu......................................................................  2     1   Mm3
....................uauggcaccguGgaauucacu......................................................................  1     1   Mm3
....................uauggcaccguagaauAcacu......................................................................  2     1   Mm3
....................uauggcaccUuagaauucacu......................................................................  1     1   Mm3
....................uauggcaccguagaauucacG......................................................................  3     1   Mm3
....................uauggcaccguagGauucacu......................................................................  1     1   Mm3
....................uauggcaccguagaauuUacu......................................................................  5     1   Mm3
....................uauggcaccgCagaauucacu......................................................................  1     1   Mm3
....................uauggUaccguagaauucacu......................................................................  1     1   Mm3
....................uauggcaccguagaauuAacu......................................................................  3     1   Mm3
....................uauggcaccguUgaauucacug.....................................................................  1     1   Mm3
....................uauggcaccguagaauucacuU.....................................................................  4     1   Mm3
....................uauUgcaccguagaauucacug.....................................................................  1     1   Mm3
....................uauggcaccgCagaauucacug.....................................................................  4     1   Mm3
....................uauggcaccguagaauucacuA.....................................................................  1     1   Mm3
....................Nauggcaccguagaauucacug.....................................................................  1     1   Mm3
....................uauggcacUguagaauucacug.....................................................................  4     1   Mm3
....................uauggUaccguagaauucacug.....................................................................  1     1   Mm3
....................uauggcaccguagaauucacGg.....................................................................  4     1   Mm3
....................uGuggcaccguagaauucacug.....................................................................  2     1   Mm3
....................uauggcaccguagaauGcacug.....................................................................  6     1   Mm3
....................uauggNaccguagaauucacug.....................................................................  1     1   Mm3
....................uauggcNccguagaauucacug.....................................................................  1     1   Mm3
```

Mature Star

```
ucaucucugcuuacaagagcuauggcaccguagaauucacuguguuuuuuucaguggguuguucggugcuguugcuuuugauguagauagcugcuguugucacuugugauuc
....................uauggcaccguagaauucacug......................................................................    2731    0    Mm3
....................uauggcaccgAagaauucacug......................................................................    9       1    Mm3
....................uauggcUccguagaauucacug......................................................................    1       1    Mm3
....................uauggcaccguagaauuUacug......................................................................    15      1    Mm3
....................uauggcaccguagaauCcacug......................................................................    10      1    Mm3
....................uauggcaccguagaauuGacug......................................................................    10      1    Mm3
....................uauggcaccguaUaauucacug......................................................................    1       1    Mm3
....................uUuggcaccguagaauucacug......................................................................    1       1    Mm3
....................uauggcaccguagaUuucacug......................................................................    1       1    Mm3
....................uauAgcaccguagaauucacug......................................................................    3       1    Mm3
....................uauggcaccguCgaauucacug......................................................................    4       1    Mm3
....................uauggcaccgGagaauucacug......................................................................    6       1    Mm3
....................uaugUcaccguagaauucacug......................................................................    1       1    Mm3
....................uauggcaccguagaauAcacug......................................................................    8       1    Mm3
....................uauggcaccguagaGuucacug......................................................................    1       1    Mm3
....................uauggcaccguGgaauucacug......................................................................    2       1    Mm3
....................uauggcaccguagaauucacCg......................................................................    1       1    Mm3
....................uauggcaUcguagaauucacug......................................................................    1       1    Mm3
....................uauggcGccguagaauucacug......................................................................    2       1    Mm3
....................uauggcaccguagaCuucacug......................................................................    1       1    Mm3
....................uauggcaccAuagaauucacug......................................................................    3       1    Mm3
....................uauggcaccguagaauuAacug......................................................................    28      1    Mm3
....................uauggcaccUuagaauucacug......................................................................    1       1    Mm3
....................uauggcaccguagaauCcacugu.....................................................................    1       1    Mm3
....................uauggcaccguagaauuAacugu.....................................................................    1       1    Mm3
....................uauggcaccguagaauucacugC.....................................................................    1       1    Mm3
....................uauggcaccguagaauGcacugu.....................................................................    1       1    Mm3
....................uauggcaccguagaauucacugu.....................................................................    138     0    Mm3
....................uauggcaccgAagaauucacugu.....................................................................    1       1    Mm3
....................uauggcacUguagaauucacugu.....................................................................    1       1    Mm3
....................uauggcaccguagaauuUacugu.....................................................................    1       1    Mm3
....................uauggcaccguagaauAcacugu.....................................................................    1       1    Mm3
....................uauggcaccguagaauucacugG.....................................................................    4       1    Mm3
....................uauggcaccguagaauucacugA.....................................................................    41      1    Mm3
....................uauggcaccguagaauucacuguU....................................................................    21      1    Mm3
....................uauggcaccguagaauucacuguA....................................................................    2       1    Mm3
.....................auggcaccguagaauucacu.......................................................................    2       0    Mm3
.....................auggcaccguagaauucacug......................................................................    5       0    Mm3
.....................................................guggguuguucgguAcuguugc.....................................    1       1    Mm3
.....................................................guggguCguucggugcuguugc.....................................    1       1    Mm3
.....................................................guggguuguucggugcuguugc.....................................    18      0    Mm3
.....................................................guggguuguucggugcuguugcu....................................    3       0    Mm3
.....................................................guggguuguucggugcuguugcuu...................................    1       0    Mm3
.....................................................guggguuguucggugcuguugcuuu..................................    1       0    Mm3
...........................................................................uuuugauguagauagcugcu.................    1       0    Mm3

...................cuauggcaccguagaauucacug......................................................................    1       0    Mm1
...................cuauggcaccguCgaauucacug......................................................................    14      1    Mm1
....................uauggUaccguagaauucac.........................................................................    1       1    Mm1
....................uauggcaccguagaauucac.........................................................................    11      0    Mm1
....................uauggcaccguCgaauucac.........................................................................    22      1    Mm1
....................uauggcNccguagaauucacu.......................................................................    2       1    Mm1
....................uauggcaccguagaauuUacu.......................................................................    2       1    Mm1
....................Nauggcaccguagaauucacu.......................................................................    1       1    Mm1
....................uauggcaccguagaauuAacu.......................................................................    10      1    Mm1
....................uGuggcaccguagaauucacu.......................................................................    1       1    Mm1
....................uaGggcaccguagaauucacu.......................................................................    1       1    Mm1
....................uauggcaccguCgaauucacu.......................................................................    738     1    Mm1
....................uauggcaccgGagaauucacu.......................................................................    3       1    Mm1
....................uauggcaccguagaauucacu.......................................................................    837     0    Mm1
....................uaAggcaccguagaauucacu.......................................................................    1       1    Mm1
....................uauggcaccguagaauGcacu.......................................................................    4       1    Mm1
....................uauggcaccguagaauAcacu.......................................................................    4       1    Mm1
....................uauggcaccguagaauuGacu.......................................................................    3       1    Mm1
....................uauggcaccgAagaauucacu.......................................................................    3       1    Mm1
....................uauggcaccguagaauCcacu.......................................................................    4       1    Mm1
....................uauggcaccgAagaauucacug......................................................................    4       1    Mm1
....................uauggcaccguagaauCcacug......................................................................    5       1    Mm1
....................uauggcaccguagaCuucacug......................................................................    1       1    Mm1
....................uauggcaccguagaauuGacug......................................................................    6       1    Mm1
```

```
                    Mature                          Star
ucaucucugcuuacaagagcuauggcaccguagaauucacuguguuuuuuucaguggguuguucggugcuguugcuuuugauguagauagcugcuguugucacuugugauuc
....................uauggcaccAuagaauucacug......................................................................  1      1  Mm1
....................uauggcaccguagGauucacug......................................................................  6      1  Mm1
....................uauggcacUguagaauucacug......................................................................  8      1  Mm1
....................uauggcaccguagaauucacuA......................................................................  1      1  Mm1
....................uauggcaccguCgaauucacug......................................................................  57115  1  Mm1
....................uauggcaccguagaaCucacug......................................................................  1      1  Mm1
....................uauggcaUcguagaauucacug......................................................................  1      1  Mm1
....................uauggcGccguagaauucacug......................................................................  1      1  Mm1
....................uauggcaccguagaauAcacug......................................................................  3      1  Mm1
....................uaGggcaccguagaauucacug......................................................................  2      1  Mm1
....................uCuggcaccguagaauucacug......................................................................  1      1  Mm1
....................uauggcaccgGagaauucacug......................................................................  4      1  Mm1
....................uauggcaccguagaauuAacug......................................................................  8      1  Mm1
....................uauggcaccguagaauucacGg......................................................................  1      1  Mm1
....................uauggcaccguagaauucUcug......................................................................  1      1  Mm1
....................uauggcaccguagaauGcacug......................................................................  3      1  Mm1
....................Nauggcaccguagaauucacug......................................................................  2      1  Mm1
....................uauggcaccguagaauuUacug......................................................................  6      1  Mm1
....................uauggcaccguagaauucacug......................................................................  1779   0  Mm1
....................uauggcaccgCagaauucacug......................................................................  2      1  Mm1
....................uauggcaccguGgaauucacug......................................................................  4      1  Mm1
....................uauggcaccguagaauucacuU......................................................................  2      1  Mm1
....................uauggcaccguUgaauucacug......................................................................  12     1  Mm1
....................uauggcaccgAagaauucacugu.....................................................................  1      1  Mm1
....................uauggcaccgCagaauucacugu.....................................................................  1      1  Mm1
....................uauggcaccguagaauucacugG.....................................................................  5      1  Mm1
....................uauggcaccguagaauucacugu.....................................................................  155    0  Mm1
....................uauggcaccguagaauuAacugu.....................................................................  2      1  Mm1
....................uauggcaccgGagaauucacugu.....................................................................  1      1  Mm1
....................uGuggcaccguagaauucacugu.....................................................................  1      1  Mm1
....................uauggcaccguagaauuGacugu.....................................................................  1      1  Mm1
....................uauggcaccguCgaauucacugu.....................................................................  1172   1  Mm1
....................uauggcaccguagaauucacugA.....................................................................  86     1  Mm1
....................uauggcaccguagaauuUacugu.....................................................................  2      1  Mm1
....................uauggcaccguagaauucacuguU....................................................................  6      1  Mm1
....................uauggcaccguagaauucacugAg....................................................................  2      1  Mm1
.....................auggcaccguagaauucacu.......................................................................  1      0  Mm1
.....................auggcaccguCgaauucacug......................................................................  25     1  Mm1
......................uggcaccguCgaauucacug......................................................................  7      1  Mm1
.........................caccguCgaauucacugu.....................................................................  7      1  Mm1
.....................................................guggguuguucggugcCguugc.....................................  1      1  Mm1
.....................................................guggguuguucggugcuguugc.....................................  166    0  Mm1
.....................................................guggguuguucggugcuAuugc.....................................  1      1  Mm1
.....................................................guggguuguucggugcuUuugc.....................................  1      1  Mm1
.....................................................guggguNguucggugcuguugc.....................................  1      1  Mm1
.....................................................guggguuguucggugcGguugc.....................................  1      1  Mm1

ucaucucugcuuacaagag.............................................................................................  1      0  Mm2
...................cuauggcaccguagaauucacug......................................................................  5      0  Mm2
....................uauggcaccguagaauuc..........................................................................  11     0  Mm2
....................uauggcaccguagaauGc..........................................................................  1      1  Mm2
....................uauggcaccguagaauuca.........................................................................  4      0  Mm2
....................uauggcaccguagaauucac........................................................................  202    0  Mm2
....................uauggcaccguagaauuGac........................................................................  1      1  Mm2
....................uauggcaccgAagaauucac........................................................................  1      1  Mm2
....................uauggcaccguagaauuAac........................................................................  3      1  Mm2
....................uauggcaccgCagaauucac........................................................................  1      1  Mm2
....................uauggcGccguagaauucac........................................................................  3      1  Mm2
....................uauggcaccguagaauAcac........................................................................  1      1  Mm2
....................uauggcaccguagaauucCcu.......................................................................  1      1  Mm2
....................Cauggcaccguagaauucacu.......................................................................  1      1  Mm2
....................uauggUaccguagaauucacu.......................................................................  2      1  Mm2
....................Nauggcaccguagaauucacu.......................................................................  2      1  Mm2
....................uauggcaccgAagaauucacu.......................................................................  6      1  Mm2
....................uauggcaccguGgaauucacu.......................................................................  2      1  Mm2
....................uauggcUccguagaauucacu.......................................................................  1      1  Mm2
....................uauggcaccguagaauAcacu.......................................................................  14     1  Mm2
....................uauggcaccguagaauucacG.......................................................................  16     1  Mm2
....................uaugAcaccguagaauucacu.......................................................................  1      1  Mm2
....................uauggcaccguagaauCcacu.......................................................................  16     1  Mm2
```

```
ucaucucugcuuacaagagcuauggcaccguagaauucacuguguuuuuuucagugggguuguucggugcuguugcuuuugauguagauagcugcuguugucacuugugauuc
....................uauggcaUcguagaauucacu.........................................................................  2      1  Mm2
....................uauggcaccguaCaauucacu.........................................................................  1      1  Mm2
....................uauggcaccguagaauGcacu.........................................................................  13     1  Mm2
....................uauggcaccguagaauuUacu.........................................................................  24     1  Mm2
....................uauAgcaccguagaauucacu.........................................................................  5      1  Mm2
....................uauggcaccguUgaauucacu.........................................................................  2      1  Mm2
....................uauggcaccguagaauucacA.........................................................................  3      1  Mm2
....................uUuggcaccguagaauucacu.........................................................................  1      1  Mm2
....................uauggcaccguagaauucacu.........................................................................  5849   0  Mm2
....................uauggcaccgCagaauucacu.........................................................................  7      1  Mm2
....................uauggNaccguagaauucacu.........................................................................  2      1  Mm2
....................uauCgcaccguagaauucacu.........................................................................  1      1  Mm2
....................uauggcCccguagaauucacu.........................................................................  1      1  Mm2
....................uauggcacUguagaauucacu.........................................................................  10     1  Mm2
....................uauggcaccguaAaauucacu.........................................................................  1      1  Mm2
....................uauggcaccguagCauucacu.........................................................................  1      1  Mm2
....................uauggcaccguagaauuAacu.........................................................................  53     1  Mm2
....................uaugUcaccguagaauucacu.........................................................................  2      1  Mm2
....................uauggcGccguagaauucacu.........................................................................  5      1  Mm2
....................uaugCcaccguagaauucacu.........................................................................  1      1  Mm2
....................uGuggcaccguagaauucacu.........................................................................  2      1  Mm2
....................uauggcaccUuagaauucacu.........................................................................  2      1  Mm2
....................uauggcaccgGagaauucacu.........................................................................  8      1  Mm2
....................uauggcaccguagaauuGacu.........................................................................  37     1  Mm2
....................uauggAaccguagaauucacu.........................................................................  1      1  Mm2
....................uauUgcaccguagaauucacu.........................................................................  1      1  Mm2
....................uauggcaccAuagaauucacu.........................................................................  1      1  Mm2
....................uauCgcaccguagaauucacug.......................................................................  3      1  Mm2
....................uauggcaccguagaauucUcug.......................................................................  2      1  Mm2
....................uauggcaccguaUaauucacug.......................................................................  10     1  Mm2
....................uauAgcaccguagaauucacug.......................................................................  7      1  Mm2
....................uauggcaccguagaUuucacug.......................................................................  4      1  Mm2
....................uGuggcaccguagaauucacug.......................................................................  7      1  Mm2
....................uauggcaccguagaGuucacug.......................................................................  2      1  Mm2
....................uauggcaccguagaaCucacug.......................................................................  1      1  Mm2
....................uauggcaccAuagaauucacug.......................................................................  8      1  Mm2
....................uNuggcaccguagaauucacug.......................................................................  1      1  Mm2
....................uaGggcaccguagaauucacug.......................................................................  2      1  Mm2
....................Gauggcaccguagaauucacug.......................................................................  1      1  Mm2
....................Aauggcaccguagaauucacug.......................................................................  4      1  Mm2
....................uauggcacAguagaauucacug.......................................................................  1      1  Mm2
....................uauggcaccguagaauucacuA.......................................................................  16     1  Mm2
....................uauggNaccguagaauucacug.......................................................................  1      1  Mm2
....................Nauggcaccguagaauucacug.......................................................................  11     1  Mm2
....................uauggcaGcguagaauucacug.......................................................................  1      1  Mm2
....................uUuggcaccguagaauucacug.......................................................................  7      1  Mm2
....................uauggcaccguagaauucacCg.......................................................................  1      1  Mm2
....................uauggcaccguagaauuGacug.......................................................................  81     1  Mm2
....................uauggcaccguagaauuAacug.......................................................................  174    1  Mm2
....................uauggcaccguagUauucacug.......................................................................  3      1  Mm2
....................uauggcaccguagaauGcacug.......................................................................  43     1  Mm2
....................uauggcaccguaAaauucacug.......................................................................  1      1  Mm2
....................uaugAcaccguagaauucacug.......................................................................  3      1  Mm2
....................uauggcaccguUgaauucacug.......................................................................  5      1  Mm2
....................uauggcaccgGagaauucacug.......................................................................  21     1  Mm2
....................uauggcacUguagaauucacug.......................................................................  31     1  Mm2
....................uauggcaccguagaauucacug.......................................................................  20205  0  Mm2
....................uauggcaccguagaAucacug.......................................................................  1      1  Mm2
....................uauggcaccguagaauucaUug.......................................................................  3      1  Mm2
....................uauggcaccguagaauucacuU.......................................................................  26     1  Mm2
....................uauggcaccgAagaauucacug.......................................................................  27     1  Mm2
....................uauggcaccguagaauAcacug.......................................................................  52     1  Mm2
....................uauggcaccguagaauuNacug.......................................................................  1      1  Mm2
....................uauggcNccguagaauucacug.......................................................................  2      1  Mm2
....................uauggcaccguCgaauucacug.......................................................................  24     1  Mm2
....................uauUgcaccguagaauucacug.......................................................................  3      1  Mm2
....................uauggcaUcguagaauucacug.......................................................................  2      1  Mm2
....................uauggcaccguagaauucGcug.......................................................................  2      1  Mm2
....................uauggcaccguagaauucCcug.......................................................................  2      1  Mm2
....................uauggcGccguagaauucacug.......................................................................  15     1  Mm2
```

```
                    Mature                           Star
ucaucucugcuuacaagagcuauggcaccguagaauucacuguguuuuuuucaguggguuguucggugcuguugcuuuugauguagauagcugcuguugucacuugugauuc
....................uauggcaccguagaauucacGg......................................................................  37    1  Mm2
....................uauggUaccguagaauucacug......................................................................  12    1  Mm2
....................uauggcaccguGgaauucacug......................................................................  2     1  Mm2
....................uauggcaccUuagaauucacug......................................................................  7     1  Mm2
....................uaCggcaccguagaauucacug......................................................................  3     1  Mm2
....................uauggcaAcguagaauucacug......................................................................  2     1  Mm2
....................uaugUcaccguagaauucacug......................................................................  5     1  Mm2
....................uaugCcaccguagaauucacug......................................................................  8     1  Mm2
....................uauggcaccguagGauucacug......................................................................  1     1  Mm2
....................uauggcaccgCagaauucacug......................................................................  20    1  Mm2
....................uauggcaccguagaauucacuC......................................................................  8     1  Mm2
....................uauggcaccguagaauucacAg......................................................................  3     1  Mm2
....................uauggcaccguagaauuUacug......................................................................  97    1  Mm2
....................uauggcaccguagaauCcacug......................................................................  55    1  Mm2
....................uauAgcaccguagaauucacugu.....................................................................  1     1  Mm2
....................uauggcaccguagaauuUacugu.....................................................................  6     1  Mm2
....................uauggAaccguagaauucacugu.....................................................................  1     1  Mm2
....................uauggcaccguagaauucacugC.....................................................................  1     1  Mm2
....................uauggcaccguagaauuGacugu.....................................................................  4     1  Mm2
....................uauggcaccguagaauucacuUu.....................................................................  1     1  Mm2
....................uauggcCccguagaauucacugu.....................................................................  1     1  Mm2
....................uauggcaccguagaauGcacugu.....................................................................  1     1  Mm2
....................uauggcGccguagaauucacugu.....................................................................  2     1  Mm2
....................uauggcaccguagaauucacugA.....................................................................  205   1  Mm2
....................uaGggcaccguagaauucacugu.....................................................................  1     1  Mm2
....................uauggcaccguagaauucacugG.....................................................................  20    1  Mm2
....................uauggcaccgAagaauucacugu.....................................................................  1     1  Mm2
....................uauggcaccguagaauuAacugu.....................................................................  7     1  Mm2
....................uauggcaccguUgaauucacugu.....................................................................  1     1  Mm2
....................uauggcacUguagaauucacugu.....................................................................  2     1  Mm2
....................uauggcaccguagaauucacAgu.....................................................................  1     1  Mm2
....................uauggcaccguagaauAcacugu.....................................................................  2     1  Mm2
....................uauggcaccguagaauucacugu.....................................................................  901   0  Mm2
....................uaNggcaccguagaauucacugu.....................................................................  1     1  Mm2
....................uauggcaccguagaaCucacugu.....................................................................  1     1  Mm2
....................uauggcaccguagaauucacuguA....................................................................  4     1  Mm2
....................uauggcaccguagaauucacuguU....................................................................  19    1  Mm2
....................uauggcaccguagaauucacuguUu...................................................................  1     1  Mm2
....................uauggcaccguagaauucacugugG...................................................................  1     1  Mm2
....................uauggcaccguagaauucacuguUuuuuuuu.............................................................  1     1  Mm2
.....................auggcaccguagaauucac........................................................................  1     0  Mm2
.....................auggcaccguagaauucacu.......................................................................  5     0  Mm2
.....................auggcaccguaAaauucacu.......................................................................  1     1  Mm2
.....................auggcaccguagaauucacug......................................................................  35    0  Mm2
.....................auggcaccguagaauucGcug......................................................................  1     1  Mm2
.....................auggcaccguagaauucacugA.....................................................................  1     1  Mm2
.....................auggcaccguagaauucacugu.....................................................................  1     0  Mm2
......................uggcaccguagaauucacug......................................................................  1     0  Mm2
..........................................uguuuuuuucUguggguuguucggugcuguugc.....................................  1     1  Mm2
.....................................................guggguuguucggugcuguug......................................  2     0  Mm2
.....................................................guggguuguucggugcuAuugc.....................................  2     1  Mm2
.....................................................guggguuguucggugcuguugc.....................................  68    0  Mm2
.....................................................guggguuguucggugcuguugcG....................................  1     1  Mm2
.....................................................guggguuguucggugcuguugcu....................................  5     0  Mm2
.....................................................guggguuguucggugcuguugcA....................................  2     1  Mm2
.....................................................guggguuguucggugcuguugcuu...................................  1     0  Mm2
```
